# Transcutaneous Electrical Acupoint Stimulation Decreases the Incidence of Postoperative Nausea and Vomiting After Laparoscopic Non-gastrointestinal Surgery: A Multi-Center Randomized Controlled Trial

**DOI:** 10.3389/fmed.2022.766244

**Published:** 2022-03-14

**Authors:** Wei Gao, Linzhong Zhang, Xuechang Han, Lai Wei, Jie Fang, Xiaqing Zhang, Jiaqiang Zhang, Haiyun Wang, Qi Zhou, Chenggang Wang, Wenting Chen, Xinli Ni, Lan Yang, Ruini Du, Ge Wang, Bingyu Liu, Yajuan Li, Shanshan Zhang, Qiang Wang

**Affiliations:** ^1^Department of Anesthesiology, Center for Brain Science, The First Affiliated Hospital of Xi'an Jiaotong University, Xi'an, China; ^2^Department of Anesthesiology, Second Hospital of Shanxi Medical University, Taiyuan, China; ^3^Department of Anesthesiology, The First Affiliated Hospital, College of Clinical Medicine of Henan University of Science and Technology, Luoyang, China; ^4^Department of Anesthesiology, Hunan Provincial People's Hospital, Changsha, China; ^5^Department of Anesthesiology, The First Affiliated Hospital of Henan University of Chinese Medicine, Zhengzhou, China; ^6^Department of Anesthesiology, Affiliated Hospital of Shaanxi University of Chinese Medicine, Xianyang, China; ^7^Department of Anesthesiology, Henan Provincial People's Hospital, Zhengzhou, China; ^8^Department of Anesthesiology, The Third Central Hospital of Tianjin, Tianjin, China; ^9^Department of Anesthesiology, The First Hospital of Hunan University of Chinese Medicine, Changsha, China; ^10^Department of Anesthesiology, Shanxi Traditional Chinese Medicine Hospital, Taiyuan, China; ^11^Department of Anesthesiology, Shuguang Hospital Affiliated to Shanghai University of Traditional Chinese Medicine, Shanghai, China; ^12^Department of Anesthesiology, General Hospital of Ningxia Medical University, Yinchuan, China

**Keywords:** antiemetic drugs, laparoscopic non-gastrointestinal surgery, nausea, transcutaneous electrical stimulation, vomiting

## Abstract

**Importance:**

Postoperative nausea and vomiting (PONV) gives patients a bad experience and negates their good recovery from surgery.

**Objective:**

This trial aims to assess the preventive effectiveness of transcutaneous electrical acupoint stimulation (TEAS) on the incidence of PONV in high-risk surgical patients.

**Design:**

The large sample size, multicenter, evaluator-blinded, and randomized controlled study was conducted between September 3, 2019 to February 6, 2021.

**Setting:**

The 12 hospitals were from different Chinese provinces.

**Participants:**

After obtaining ethics approval and written informed consent, 1,655 patients with Apfel score ≥ 3 points were enrolled for selective laparoscopic non-gastrointestinal surgery under general anesthesia.

**Interventions:**

Patients were randomly allocated into the TEAS and Sham group with a 1:1 ratio. The TEAS group was stimulated on bilateral Neiguan and Zusanli acupoints after recovery from anesthesia on the surgical day and the next morning for 30 min, while the Sham group received an identical setting as TEAS but without currents delivered. Electronic patient self-reported scale was used to evaluate and record the occurrence of PONV.

**Main Outcomes and Measures:**

Primary clinical end point is the incidence of PONV which was defined as at least one incidence of nausea, retching, or vomiting after operation within postoperative 24 h.

**Results:**

Compared with the Sham treatment, the TEAS lowered the PONV incidence by 4.8% (29.4 vs. 34.2%, *P* = 0.036) and vomiting incidence by 7.4% (10.4 vs. 17.8%, *P* < 0.001). TEAS also lowered persistent nausea incidence and PONV scores and decreased PONV related complications and Quality of Recovery−40 scores (*P* < 0.05). TEAS lowered the 24 h PONV risk by 20% (OR, 0.80, 95% CI, 0.65 −0.98; *P* = 0.032), and lowered hazard ratio by 17% (HR, 0.83, 95% CI, 0.70–0.99; *P* = 0.035). Both TEAS and palonosetron were the independent PONV risk protective factors for 24 h PONV incidence and cumulative PONV incidence. The combination of TEAS and palonosetron was the most effective strategy to reduce the PONV incidence (*P* < 0.001).

**Conclusions and Relevance:**

TEAS attenuated the PONV incidence and severity in high-risk surgical patients and may be applied clinically as a complement therapy to prevent PONV.

**Clinical Trial Registration:**

https://clinicaltrials.gov/ct2/show/NCT04043247, identifier: NCT04043247.

## Key Points

- **Question:** Dose transcutaneous electrical stimulation prevent postoperative nausea and vomiting (PONV) in high-risk surgical patients?
- **Findings:** The electrical stimulation significantly reduced the PONV incidence and its severity. Both TEAS and palonosetron were independent PONV risk protective factors for 24 h PONV incidence and cumulative PONV incidence.
- **Meaning:** TEAS may enhance recovery of PONV high-risk surgical patients and should be routinely used clinically as a complementary strategy.

## Introduction

Postoperative nausea and vomiting (PONV), characterized as nausea, retching, or vomiting or any these symptoms in combination after surgery, is one of the most common complaints after surgery with an overall incidence of 30%, but its incidence can be as high as to be 60–80% in high-risk patients who have three or four PONV risk factors (1). Nausea gives patients an extremely afflictive medical experience, induces stomach discomfort including the feeling of keck and poor appetite, and even causes dizziness and headache. Vomiting increases the potential risks of electrolyte imbalances, pain, incision dehiscence or hernia, dehydration, esophageal damage, or airway aspiration (2). PONV prevention has been included in enhanced recovery after surgery (ERAS) management strategy. Even though the incidence of PONV can be decreased by using less opioids and inhalational anesthetics and even using antiemetic drugs, its occurrence is still high, with up to 20% of high-risk patients treated with three antiemetic prophylaxis (1, 3).

Owning to multiple mechanisms triggering PONV, several antiemetic drugs are often used clinically in high-risk PONV patients (4), but their effectiveness is still limited and include unwanted side effects such as headaches, xerostomia, abnormal liver function, and extrapyramidal reactions (5–8). Compared to antiemetic drugs, non-pharmaceutical therapy including acupuncture has considerable advantages, for example, non-toxic effects, which has been widely used in postoperative pain analgesia and gastrointestinal function rehabilitation (9, 10). Indeed, acupuncture, a safe and effective non-pharmacotherapy, can directly or indirectly inhibit the emesis center or regulate gastrointestinal function through multiple regulatory mechanisms, such as modulating gastric motility through somatovisceral reflex and influencing the endogenous opioid system and serotonin transmission (11–14). Acupoint Neiguan (P6) stimulation was reported to be comparable to antiemetic drugs in reducing PONV incidence but its effectiveness in combination with antiemetic drugs is inconclusive (1). Transcutaneous electrical acupoint stimulation (TEAS) with transcutaneous electrical nerve stimulation applied to acupuncture points has been used to prevent PONV occurrence (15, 16). However, its effectiveness in preventing PONV has not been established yet due to small sampling size and being not well controlled in the previous studies (1). Therefore, the large sample size, multicenter, evaluator-blinded, and randomized controlled study was carried out to verify the effectiveness of TEAS on P6 and Zusanli (ST36) in reducing the incidence of PONV.

## Methods

### Study Design and Participants

This multicenter randomized controlled trial was approved by the Ethics Committee of the First Affiliated Hospital of Xi'an Jiaotong University (XJTU1AF2019LSK-084) and then registered at ClinicalTrials.gov (NCT04043247). The detailed trial protocol is available online as Supplementary Material 1. The study was carried out in accordance with clinical research CONSORT and acupuncture clinical trial STRICTA to provide reliable concrete evidence for clinical application of TEAS in PONV prophylaxis (Supplementary Material 2). The following inclusion criteria were included: (1) selective laparoscopic non-gastrointestinal surgery under general anesthesia; (2) age 18–50 years, BMI 15–40 kg/m^2^, ASA class I–III; (3) Apfel score ≥ 3 points; and (4) ability to understand, sign informed consent, and coordinate intervention and evaluation. The following exclusion criteria were included: (1) pregnant and breast-feeding patients; (2) TEAS contraindications such as skin allergy, breakage, infection or itching of test points, allergic to adhesive tape, pacemaker implanted; (3) history of alcohol, opioids, or other drug abuse; (4) likely admission to ICU after surgery; and (5) participation in other clinical studies within 3 months before admission to this study. All investigators were trained according to the standardized acupoints selection and TEAS manipulation by a senior licensed acupuncturist with 20 years of practicing experience.

### Enrolment, Randomization, and Blinding

After written informed consent was obtained, patients scheduled for selective laparoscopic non-gastrointestinal surgery under general anesthesia were screened for potential enrolments from 12 hospitals (listed in the authorship) in China from September 3, 2019 to February 6, 2021. The eligible patients were randomly divided into the Sham and TEAS groups in a 1:1 ratio (with a block size of 4) by Crabyter scientific research system (Xinyu Information Technology Co., Ltd.). All patients, anesthesiologists, surgeons, and evaluators were blinded for group allocation, screening, intervention treatment, and statistical analysis. Designated evaluators who were totally blinded took charge for follow-up data collections.

### Clinical End Points

Primary clinical end point is the incidence of PONV, which was defined as nausea, retching, and/or vomiting after operation within postoperative 24 h. Secondary clinical end points were the postoperative 24 h PONV severity and the 40-item Quality of Recovery (QoR-40) and PONV-related complications. The PONV severity was assessed with the time and visual analog scale (VAS) score of first PONV, the cumulative numbers, and VAS score in postoperative 24 h. The specific contents of QoR-40 score include five aspects: physical comfort (12 items, 60 scores), emotional state (9 items, 45 scores), physical independence (5 items, 25 scores), psychological support (7 items, 35 scores), and pain (7 items, 35 scores). PONV-related complications are dizziness and headache, electrolyte imbalances, and pain.

### Anesthesia and Surgery

After receiving 0.5 mg pre-medication of anticholinergics drug penehyclidine, atropine, or scopolamine, patients were inducted with propofol (2 mg/kg), sufentanil (0.3 μg/kg), and cisatracurium (0.15 mg/kg), and maintained with remifentanil (0.1 μg/kg/h), cisatracurium (0.1 mg/kg/h), dexmedetomidine (initial dose 1 μg/kg for 10 min, maintenance dose 0.4 μg/kg/h), and sevoflurane (1% in 2 L/min enriched oxygen). Propofol (4–8 mg/kg/h) as necessary was used for anesthesia maintenance to keep BIS 40–60 throughout surgery. Patients were treated with 50 mg non-steroidal anti-inflammatory drug flurbiprofen axeril or 40 mg parecoxib at the end of surgery, and were intravenous injection pumped with sufentanil individualized postoperative analgesia pump to keep the perioperative pain numerical score at <4 points.

### Interventions

Both groups received 5 mg dexamethasone (before induction) combined with 0.075 mg palonosetron (before induction) as the first choice. When palonosetron was not available at some hospitals, patients received 5 mg dexamethasone (before induction) combined with 2 mg tropisetron (at the end of surgery) instead. According to the criteria of WHO acupuncture points, P6 and ST36 were selected in this study (Supplementary Figure 1). These acupoints are far from the operation site. P6 is located at 2 inches above palm wrist transverse striation, between the palm long tendon and the temporal flexor tendon. ST36 is located at 3 inches below EX-LE5 (the lateral pit of the patella and patellar ligament), and one transverse finger outward lateral tibia. The true or sham TEAS was given to the TEAS or sham group patients, respectively, in the PACU when recovered from anesthesia (to be awake and calm, Richmond Agitation-Sedation Scale = 0) on the same surgical day and on the next morning of surgical ward for 30 min. The bilateral P6 and ST36 were pressed to achieve a tingling sensation called “de qi”, and electrode slices were stuck and fixed on those acupoints. Interveners connected electrode slices to electrical stimulator (TEAS stimulator, SDZ-V; Hwato, China) and selected the distant-dense wave with the frequency of 2/10 Hz. After adjusting the intensity to the patient's maximum tolerance, the treatment lasted for 30 min. Patients in the sham group received identical settings and manipulation to the TEAS group, except that the wire between electrode pads and TEAS stimulator was cut so that no current could be delivered. The 5-HT_3_ receptor antagonists or propofol or haloperidol were used for PONV rescue when necessarily.

### Data Collection

Electronic patient self-reported scale (Jiangsu Rehn Medtech Technology Co., Ltd.) was used to evaluate and record the occurrence of PONV, including time and corresponding severity. The severity was evaluated with VAS score which is a psychometric response scale to measure the intensity of subjective characteristics or attitudes and has been widely used for pain quantification. In our study, the severity of nausea and vomiting was rated on the VAS score from 0 to 10 (0 = no nausea or vomiting, 10 = maximum severity). Once patients suffered PONV, patients or their caregivers pressed the buttons on an electronic scale and reported the related information (Supplementary Figure 2). Postoperative 24 h cumulative PONV incidence refers to the cumulative number of PONV episodes in the postoperative 24 h period. Persistent nausea was defined as continuous nausea lasting over 5 min. To avoid the leakage of allocation, evaluators went to the wards after 12 a.m. at the 1st and 2nd postoperative day to check the electronic patient self-reported system running condition and obtain the information of QoR-40 and complications. The postoperative 30-day complications and adverse events were obtained through telephone follow-ups and were stored in the electronic medical record system.

### Statistical Analysis

#### Power Calculation

In previous studies, the PONV incidence was reported to be ~40%, and the PONV incidence was reduced by 7% with the TEAS treatment (15). With significance set at 0.05 and power set at 80%, the sample size required to detect differences was 742 patients in each group calculated with the Pass 11 software (NCSS, LLC. Kaysville, Utah, USA). Taking into account a loss-to-follow-up rate of about 10%, 1,634 patients in total were enrolled to meet the minimum sample size requirement.

Intention-to-treat analysis was used for all enrolled patients. Continuous variables were presented as mean ± SD if data are normally distributed; otherwise, they were presented as median (interquartile range, IQR). Categorical values were presented as numbers (percentages). The differences between two groups were compared using the Mann-Whitney or Chi-square tests. The Kaplan-Meier log-rank test was used to illustrate the cumulative incidence of nausea and vomiting. Variables with significant differences (*P* < 0.10) in univariate analysis were included in multivariate analysis, which was used to identify the associated risk factors of the PONV occurrence. The comparison of independent risk factors related to PONV was further analyzed by chi-square tests or Kaplan-Meier log-rank test. A 2-sided *P-*value < 0.05 was considered to be of statistical significance. All these statistical analyses were performed using the SPSS software (version 25.0, IBM Corporation, NY, USA). All authors had access to the study data and reviewed and approved the final manuscript.

## Results

### Characteristics of Study Population

**Figure 1:**
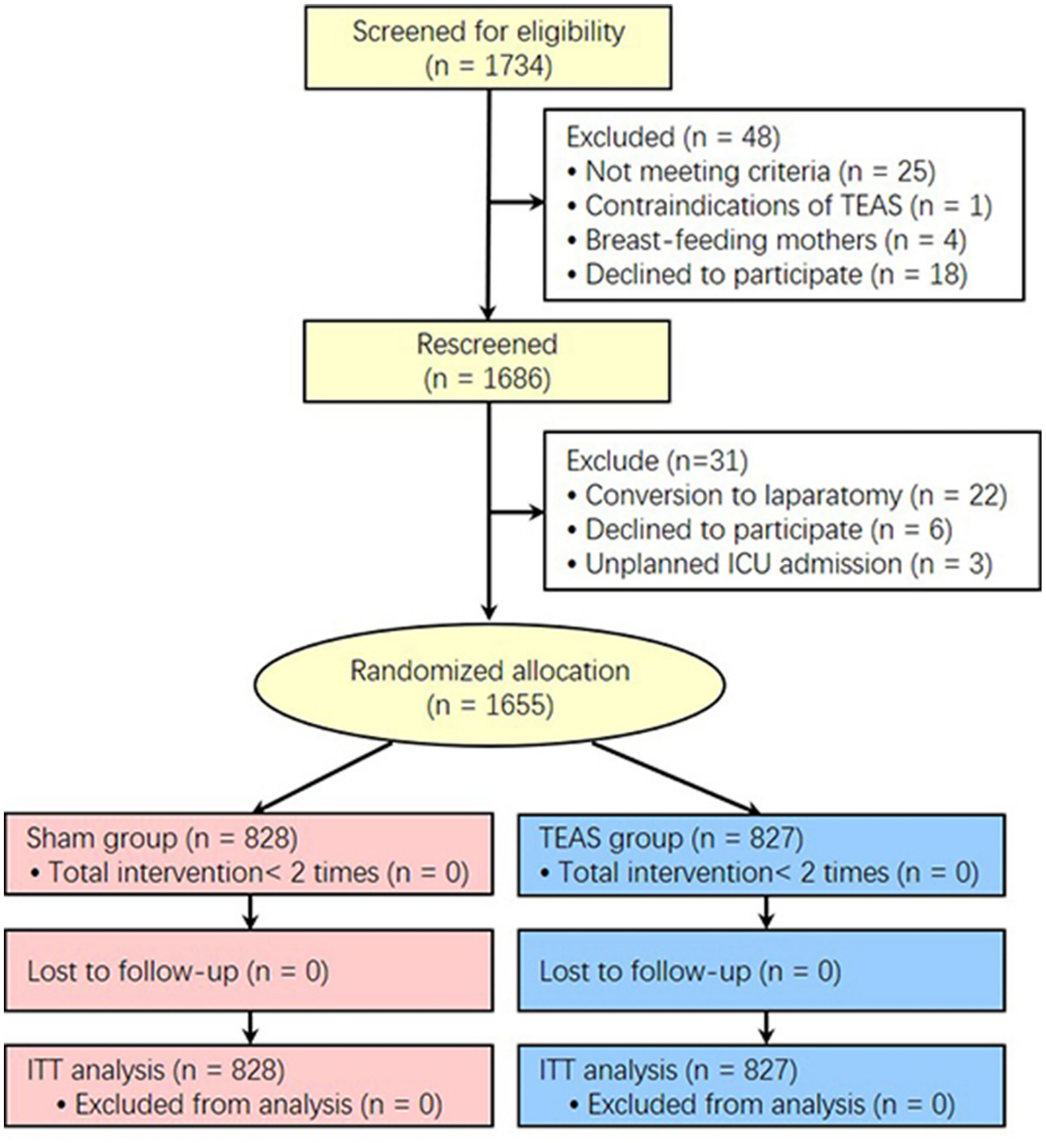
CONSORT diagram for patients enrolled in the study. TEAS, transcutaneous electrical acupoint stimulation; ITT, Intention-To-Treat.

A total of 1,734 patients were scheduled for laparoscopic non-gastrointestinal surgery at 12 hospitals in China between September 3, 2019 and February 6, 2021. At two-step recruitment screenings, 48 patients were excluded at the first screening and of those, 31 patients were excluded at the second screening. A total of 1,655 patients were enrolled and randomly divided into two groups: 828 patients allocated to the Sham group and 827 patients to the TEAS group. All 1,655 patients were included in the intention-to-treat analysis (Figure 1). No TEAS-related adverse effects occurred in the study. The demographics, comorbidities, preoperative SAS anxiety score, surgical and anesthesia data, RASS scores after extubation, and PACU electrolyte index between the TEAS and Sham group were presented in Table 1. The selective laparoscopic non-gastrointestinal surgery under general anesthesia included gynecology (>70%), cholecystectomy, urinary, hernia repairment, and liver surgery. The anticholinergics, antiemetics, and multimode analgesics used during the perioperative period showed no significant differences between the two groups, except the parecoxib usage was more often higher in the TEAS group than in the Sham group (Table 2). The patients with or without parecoxib had no significant difference of PONV incidences.

**Table 1:** Baseline characteristics.

	**TEAS**	**Sham**	***P*-value**
	**(*n* = 827)**	**(*n* = 828)**	
Age, yr	39.0 (31.0, 46.0)	39.0 (31.0, 46.0)	0.693
BMI, kg/m^2^	23.1 (21.0, 25.4)	22.7 (20.6, 24.8)	0.001
Education level, *n* (%)			0.154
≤ Primary education	223 (27.6)	209 (25.6)	
Secondary education	147 (18.2)	179 (22.0)	
Tertiary education	439 (54.3)	427 (52.4)	
**Apfel's score**, ***n*** **(%)**			
Female	803 (97.1)	803 (97.0)	0.888
Non-smoker	821 (99.3)	818 (98.8)	0.316
Use of postoperative opioids	734 (88.8)	737 (89.0)	0.869
History of PONV or motion sickness	477 (57.7)	485 (58.6)	0.712
4 scores	354 (42.8)	359 (43.4)	0.821
**Comorbidities**, ***n*** **(%)**			
Coronary heart disease	3 (0.4)	5 (0.6)	0.479
Stroke	2 (0.2)	1 (0.1)	0.563
Diabetes	10 (1.2)	5 (0.6)	0.194
Hypertension	40 (4.8)	56 (6.8)	0.094
SAS anxiety score	40.0 (32.0, 45.0)	40.0 (32.0, 45.0)	0.942
The operation types, *n* (%)			0.147
Gynecology	610 (73.8)	587 (70.9)	
Cholecystectomy	142 (17.2)	145 (17.5)	
Urinary	53 (6.4)	57 (6.9)	
Hernia repairment	14 (1.7)	28 (3.4)	
Liver surgery	8 (1.0)	11 (1.3)	
Operation time, hr	1.6 (1.1, 2.3)	1.6 (1.1, 2.3)	0.373
Anesthesia time, hr	2.0 (1.5, 2.8)	2.0 (1.4, 2.8)	0.503
RASS score after extubation	0.0 (0.0, 0.0)	0.0 (0.0, 0.0)	0.144
**PACU electrolyte index**			
Na^+^, mmol/L	139.0 (137.0, 141.0)	140.0 (137.9, 141.0)	0.734
K^+^, mmol/L	3.8 (3.5, 4.0)	3.8 (3.6, 4.1)	0.512
CI^−^, mmol/L	109.0 (107.0, 111.0)	109.0 (108.0, 111.0)	0.810
Glu, mmol/L	6.3 (5.4, 7.4)	6.1 (5.3, 7.2)	0.111

*ASA, American society of anesthesiologists physical status classification system; RASS, Richmond agitation-sedation scale; PACU, post anesthesia care unit*.

*Data are presented as median (IQR) or number (%)*.

**Table 2:** Anticholinergics drugs, antiemetics, and analgesic drug records.

	**TEAS**	**Sham**	***P*-value**
	**(*n* = 827)**	**(*n* = 828)**	
Anticholinergics drugs, *n* (%)	461 (55.7)	438 (52.9)	0.245
Penehyclidine	393 (47.5)	383 (46.3)	0.606
Atropine	68 (8.8)	61 (7.6)	0.389
Scopolamine	4 (0.5)	3 (0.4)	0.669
**Prophylaxis antiemetic**, ***n*** **(%)**			
Dexamethasone	770 (93.1)	758 (91.5)	0.233
Palonosetron	431 (52.1)	438 (52.9)	0.750
Tropisetron	396 (47.9)	390 (47.1)	0.750
Dexamethasone+ Palonosetron	407 (49.2)	417 (50.4)	0.640
Dexamethasone+ Tropisetron	363 (43.9)	341 (41.2)	0.265
Rescue medication, *n* (%)	41 (5.0)	36 (4.3)	0.556
Nerve blocking, *n* (%)	81 (9.8)	70 (8.5)	0.344
NSAIDs or cyclooxygenase-2 inhibitors, *n* (%)	274 (33.1)	275 (33.2)	0.972
Flurbiprofen axetil	234 (30.2)	251 (31.2)	0.658
Parecoxib	40 (5.0)	24 (2.9)	0.034
Analgesic pump sufentanil, *n* (%)	597 (72.2)	588 (71.0)	0.596
24 h analgesic pump, μg	33.0 (0.0, 50.0)	30.0 (0.0, 60.0)	0.197
**Intraoperative medication**			
Sufentanil, μg	30 (30, 40)	30 (30, 40)	0.580
Remifentanil, μg	1,000 (600, 1,200)	1,000 (600, 1,255)	0.908
Propofol, mg	450 (300, 600)	450 (300, 600)	0.936
Sevoflurane, ml	20 (15, 30)	20 (15, 30)	0.798
**Intraoperative fluid, ml**			
Crystalloid solutions	1,000 (600, 1,500)	1,000 (600, 1,500)	0.149
Solute solutions	500 (500, 500)	500 (500, 500)	0.855

*Data are presented as median (IQR) or number (%)*.

### Primary and Secondary Outcomes

Compared to the Sham group, patients in the TEAS group had significantly lower PONV incidence (29.4 vs. 34.2%, *P* = 0.036) and lower vomiting incidence (10.4 vs. 17.8%, *P* < 0.001). For nausea, the TEAS group showed lower persistent nausea incidence (2.2 vs. 5.1%, *P* = 0.003), lower first-time nausea score, and 24 h nausea highest scores (*P* < 0.001). Specifically, in 827 patients of the TEAS group, 29.4% (243) patients suffered nausea, including 0.7% (6) patients who suffered both persistent nausea and vomiting, 1.5% (12) patients who suffered persistent nausea without vomiting, 9.7% (80) patients who suffered vomiting without persistent nausea, and 17.5% (145) patients who suffered nausea without persistent nausea or vomiting. Correspondingly, in 828 patients of the Sham group, these incidences were, respectively, 2.1% (17), 3.0% (25), 15.7% (130), and 13.4% (111) (Supplementary Figure 3).

In the vomiting patients, the TEAS group showed lower first-time vomiting score (*P* = 0.005) and lower highest vomiting scores (*P* = 0.008) together with vomiting times at the postoperative 24 h (*P* = 0.001). TEAS also significantly decreased the PONV-related postoperative 30 days complications including dizziness (*P* = 0.038), headache (*P* = 0.027), and postoperative 24 h Pain (*P* = 0.006) (Table 3). Compared to the non-PONV patients, the PONV patients had worse QoR-40 scores in all the five items (all *P* < 0.001). Compared to the 24 h QoR-40 scores in the Sham group, patients in the TEAS group had better physical comfort (*P* = 0.003), emotional state (*P* = 0.001), psychological support (*P* = 0.002), and pain (*P* = 0.012). Compared to the vomiting patients, the non-vomiting patients had significantly better scores in the same items (all *P* < 0.001) (Supplementary Table 1).

**Table 3:** PONV outcomes and related complications.

**Outcomes**	**TEAS**	**Sham**	***P-*value**
	**(*n* = 827)**	**(*n* = 828)**	
24 h PONV, *n* (%)	243 (29.4)	283 (34.2)	0.036
Vomiting, *n* (%)	86 (10.4)	147 (17.8)	< .001
**Nausea**			
Persistent nausea, *n* (%)			0.003
Persistent nausea	18 (2.2)	42 (5.1)	
Non-persistent nausea	225 (27.2)	241 (29.1)	
Non-PONV	584 (70.6)	545 (65.8)	
First-time nausea score	1.2 (2.3)	1.65 (2.8)	< .001
24 h nausea highest score	1.3 (2.4)	1.7 (2.9)	< .001
**Vomiting**			
First-time vomiting score	0.6 (1.8)	0.9 (2.0)	0.005
24 h vomiting highest score	0.6 (2.0)	0.9 (2.1)	0.008
24 h vomiting times	0.3 (0.9)	0.4 (1.2)	0.001
**Related complications**			
Dizzy, *n* (%)	321 (38.8)	363 (43.8)	0.038
Headache, *n* (%)	37 (4.5)	58 (7.0)	0.027
Electrolyte disturbance, *n* (%)	14 (1.7)	20 (2.4)	0.300
24 h pain VAS score	2.0 (1.7)	2.2 (1.8)	0.006

*PONV, Postoperative nausea and vomiting; VAS, visual analog scale score*.

*Data are presented as mean (SD) or patient's number (%)*.

### Multivariate Binary Logistic and COX Regression Analysis for PONV

Both TEAS and palonosetron were independently protective factors of PONV (Table 4). Compared with the Sham group, the TEAS group had 20%lower risk of PONV (OR, 0.80, 95% CI, 0.65–0.98; *P* = 0.032) and 48%lower risk of vomiting occurrence (OR, 0.52, 95% CI, 0.39–0.70; *P* < 0.001) by multivariate logistic regression analysis; the TEAS group delayed PONV occurrence time by 17% (HR, 0.83, 95% CI, 0.70–0.99; *P* = 0.035) and delayed vomiting occurrence time by 43% (HR, 0.57, 95% CI, 0.43–0.74; *P* < 0.001) by COX regression analysis (Supplementary Figure 4). Compared with the tropisetron antiemetic, palonosetron reduced risk of PONV by 38% (OR, 0.62, 95% CI, 0.50–0.77; *P* < 0.001) by multivariate logistic regression analysis; the palonosetron delayed postoperative 24 h PONV occurrence time by 32% (OR, 0.68, 95% CI, 0.57–0.81; *P* < 0.001) by COX regression analysis.

**Table 4:** Multivariates binary logistic and cox regression associated with PONV.

**Variable**	**Multivariate logistic**		**Cox regression**	
	**OR (95% CI)**	***P-*value**	**HR (95% CI)**	***P-*value**
**PONV**				
TEAS	0.80 (0.65, 0.98)	0.032	0.83 (0.70, 0.99)	0.035
Palonosetron	0.62 (0.50, 0.77)	<0.001	0.68 (0.57, 0.81)	<0.001
**Vomiting**				
TEAS	0.52 (0.39, 0.70)	<0.001	0.57 (0.43, 0.74)	<0.001
Palonosetron	0.31 (0.23, 0.42)	<0.001	0.34 (0.26, 0.45)	<0.001

**Figure 2:**
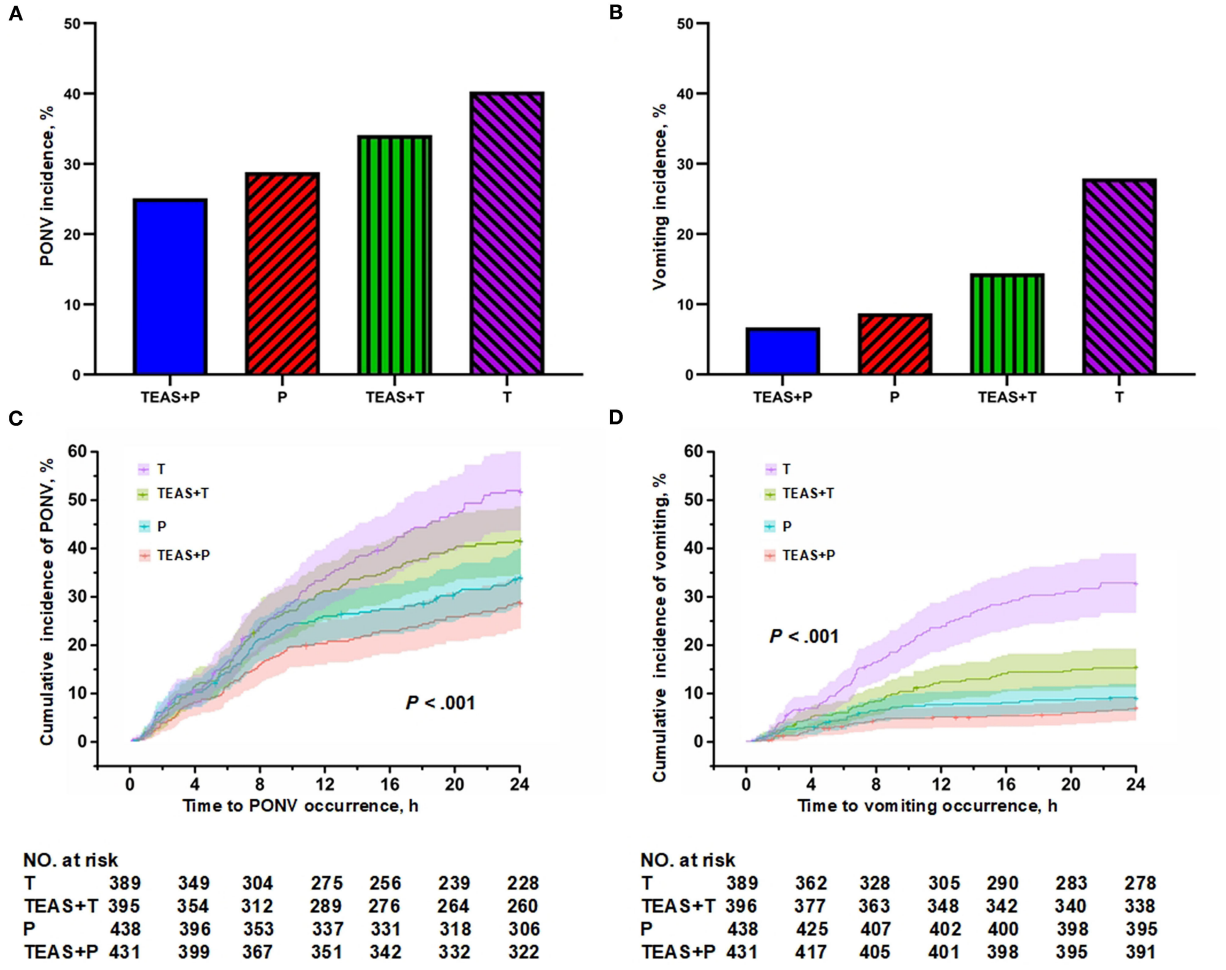
The postoperative 24 h **(A)** PONV incidences, **(B)** vomiting incidences, postoperative 24 h cumulative **(C)** PONV and **(D)** vomiting incidences among combined TEAS and palonosetron (TEAS + P), combined Sham and palonosetron (Sham + P), combined TEAS and tropisetron (TEAS + T), and combined Sham and tropisetron (Sham + T).

The combined TEAS and palonosetron group had the lowest PONV incidence (25.1%), followed with combined Sham and palonosetron (28.8%) and combined TEAS and tropisetron (34.1%), and the combined Sham and tropisetron group had the highest PONV incidence (40.3%) (*P* < 0.001). The similar distribution of cumulative PONV incidences and vomiting among the four groups was noted (Figure 2).

## Discussion

Our study found that the TEAS significantly decreased the 24 h PONV incidence and also lowered persistent nausea incidence, PONV scores, and postoperative 24 h cumulative PONV incidences. Further, the TEAS also significantly decreased PONV-related complications, and improved postoperative 24 h physical comfort and emotional state, psychological support scores, and pain score in 24 h QoR-40 Scores. We also found that both TEAS and palonosetron were the independent PONV protective factors for 24 h PONV incidence and cumulative PONV incidence.

PONV is triggered by the nerve projected to the vomiting center, which is influenced by the cerebral cortex, vestibular and cerebellar nuclei, and chemoreceptor trigger band (4). The surface of the chemoreceptor trigger band covers various receptors, such as 5-HT_3_, 5-HT_4_, opioid, cholinergic, cannabis, and dopamine receptor (17). It has been reported that the incidence of PONV can be decreased by 26% for each additional type of antiemetic drug. The incidence of PONV was 52, 37, 28, and 22% in the absence or presence of 1, 2, and 3 types of antiemetic drugs, respectively (3). The Enhanced Recovery after Surgery Society recommended that more than two types of antiemetics should be used for patients undergoing gynecologic procedures (18), while three types of antiemetics did not show improved efficacy over the two agents (19). Thus, two types antiemetics in combination (dexamethasone with tropisetron/palonosetron) were used in our study. Compared to the first-generation 5-HT_3_ receptor antagonists of tropisetron, the second-generation 5-HT_3_ receptor antagonist palonosetron has a higher binding affinity and longer half-life and duration of action, and a single injection of a 0.075 mg dose of palonosetron for PONV prevention was used for up to 24 h after surgery (20).

However, the combination of two types of antiemetic drugs only inhibited part of the PONV-related receptors, and the prophylaxis effect reached the maximum effect and increasing their doses did not increase antiemetic effects due to receptor occupancy saturation. However, the side effects were increased (7). Correspondingly, the TEAS can modulate comprehensive PONV triggering receptors and reflex pathways to prevent PONV. For example, TEAS stimulates neuronal pathways from the soma splanchnic neurons to the paraventricular nucleus of the hypothalamus (21); TEAS also reduces secretion of 5-HT in the duodenum and suppresses the activation of the nucleus tractus solitarii in the brain-stem (22). TEAS inhibits sympathetic nerve and stimulates parasympathetic nerve and hence increases the activity of acetylcholinesterase (23); TEAS also stimulates receptors such as NO, CCK-A, cannabinoid, opioid receptor, and others and regulates neurotransmitters such as serotonin, GABA, and catecholamines (14). All these result in a decreased incidence of PONV.

Furthermore, the PONV prophylaxis outcome of TEAS might be due to stimulating acupoints of P6 and ST3. P6 is currently recognized as the standard acupoint for the prevention of PONV (24), and its effect was comparable to that of antiemetic drugs (25). Stimulation of P6 and ST36 in laparoscopic radical gastrectomy for gastric cancer significantly reduced the incidence of PONV, decreased early postoperative pain intensity and analgesic dosage, shortened the time of exhaust and excretion, promoted the recovery of gastrointestinal function, and improved patient satisfaction (26). Stimulation of P6 alone or combined with ST36 for 30 min before the lumbar anesthesia of cesarean section decreased PONV, and the mechanism is related to the reduction of plasma 5-HT5 concentration (27). Electroacupuncture at P6 and St36 together was reported to decrease period-dominant frequency in the electrogastrograph, and this effect was abolished by naloxone, indicating a central opioid pathway involvement (12).

It is worth noting that we enrolled the PONV high risk patients who had Apfel ≥ 3. The Apfel score includes female gender, non-smoker, history of motion sickness or PONV, and postoperative opioid use. In our study, 97.0% of patients were female. Besides accounting for a quarter of Apfel score, females were more likely to be non-smoking (28) and have motion sickness than males (29). This agreed with the previous epidemiological study, in which females accounted for 93.3% in Apfel score 3 population and 100% in Apfel score 4 population (30). Without any PONV prophylaxis, the estimated probability of PONV in Apfel ≥ 2 patients were between 39 and 78%, whereas if Apfel ≥ 3 is present, it may rise up to 78% (1). In addition, we enrolled high PONV risk surgical types. The laparoscopic gynecological operation and the laparoscopic cholecystectomy accounts for about 70 and 18% in our study, respectively (Table 1). Without any PONV prophylaxis, the PONV incidence of gynecological surgery and cholecystectomy surgery were 69 and 59.6% (31). When PONV prophylaxis with dexamethasone and tropisetron, the PONV incidence of laparoscopic gynecological surgery had been reported even up to 77.4% (32). In contrast, we found that the PONV incidence was much lower. What factors caused these discrepancies between previous studies and the current study remain unknown. However, the study heterogeneity was avoided in our study which may be one of reasons; for example, almost all patients were female and non-smokers and the SAS anxiety score were comparable among patients.

### Limitations

The strengths of our trial are its large sample size, multiple centers, high PONV risk patients and surgeries, and the effectiveness of TEAS group in reducing PONV was clearly demonstrated. However, the unavoidable limitation in our study was that patients cannot be blinded to the intervention totally as they can sense the acupoint stimuli in the TEAS group whilst others in the sham group did not. As such, any “placebo” effects of TEAS are unknown. In addition, the balance anesthesia as a potential factor to reduce PONV, the non-standardization of anesthesia management, and the use of different regimens to prevent PONV during surgery may cause potential bias on the results. Antiemetic prophylactic medication used during our study remained similar but not identical due to different medical supplies in various centers. We have narrowed 5-HT_3_ receptor antagonists down to two specific drugs (palonosetron or tropisetron). Meanwhile, only 92.3% patients received dexamethasone, although there is no significant difference regarding dexamethasone usage between the TEAS group and Sham group (Table 2). Lastly, the sample size calculation was based on the previous publications without any antiemetic prophylaxis medication (15) which might induce sample size bias. However, our study is a large sample size and a multi-centers trial and, therefore, all those limitations were unlikely to have affected our conclusions.

## Conclusions

This multi-center randomized controlled study concluded that the use of postoperative TEAS (at anesthesia recovery arrival and in the following morning) in conjunction with antiemetic prophylaxis may significantly reduce PONV incidence and its severity. Both TEAS and palonosetron were independent PONV risk protective factors for 24 h PONV incidence and cumulative PONV incidence. The combination of TEAS and palonosetron was the most effective strategy to reduce the PONV incidence. Our work may suggest that one should consider implementing TEAS clinically for patients' better and smoother postoperative recovery.

## Data Availability Statement

The original contributions presented in the study are included in the article/Supplementary Material, further inquiries can be directed to the corresponding author/s.

## Ethics Statement

The studies involving human participants were reviewed and approved by the Ethics Committee of the First Affiliated Hospital of Xi'an Jiaotong University (XJTU1AF2019LSK-084). The patients/participants provided their written informed consent to participate in this study.

## Author Contributions

WG and QW: study conception, critically revised the manuscript for important intellectual content, and obtained funding. WG, LZ, XH, LW, JF, XZ, JZ, HW, QZ, CW, WC, XN, LY, RD, GW, and BL: acquisition or interpretation of data. WG and YL: statistical analyses. WG, LY, and QW: drafted the manuscript. WG, LZ, XH, LW, JF, XZ, JZ, HW, QZ, CW, WC, XN, LY, RD, GW, BL, and QW: provided administrative, technical, or material support.

## Funding

This study was supported by the Clinical Research Award of the First Affiliated Hospital of Xi'an Jiaotong University, China (No. XJTU1AF2021CRF-012), the National Nature Science Foundation of China (Nos. 81971290 and 81771485), and the Key Research and Development Program of Shaanxi Province (2020SF-136).

## Conflict of Interest

The authors declare that the research was conducted in the absence of any commercial or financial relationships that could be construed as a potential conflict of interest.

## Publisher's Note

All claims expressed in this article are solely those of the authors and do not necessarily represent those of their affiliated organizations, or those of the publisher, the editors and the reviewers. Any product that may be evaluated in this article, or claim that may be made by its manufacturer, is not guaranteed or endorsed by the publisher.

## Abbreviations

PONV: Postoperative nausea and vomiting
TEAS: transcutaneous electrical acupoint stimulation
QoR-40: the 40-item Quality of Recovery
P6: Neiguan
ST36: Zusanli
EX-LE5: Xiyan
5-HT3: 5-hydroxytryptamine 3
PACU: postoperative recovery unit
VAS: visual analog scale score.
