# Supplementary material for: Transcutaneous Electrical Acupoint Stimulation Decreases the Incidence of Postoperative Nausea and Vomiting After Laparoscopic Non-gastrointestinal Surgery: A Multi-Center Randomized Controlled Trial
Source: Front Med (Lausanne). 2022 Mar 14;9:766244. doi: 10.3389/fmed.2022.766244 (PMC8964119; doi:10.3389/fmed.2022.766244)
Supplement: Supplementary file 1 [file Data_Sheet_1.docx]

**Online supplemental materials**


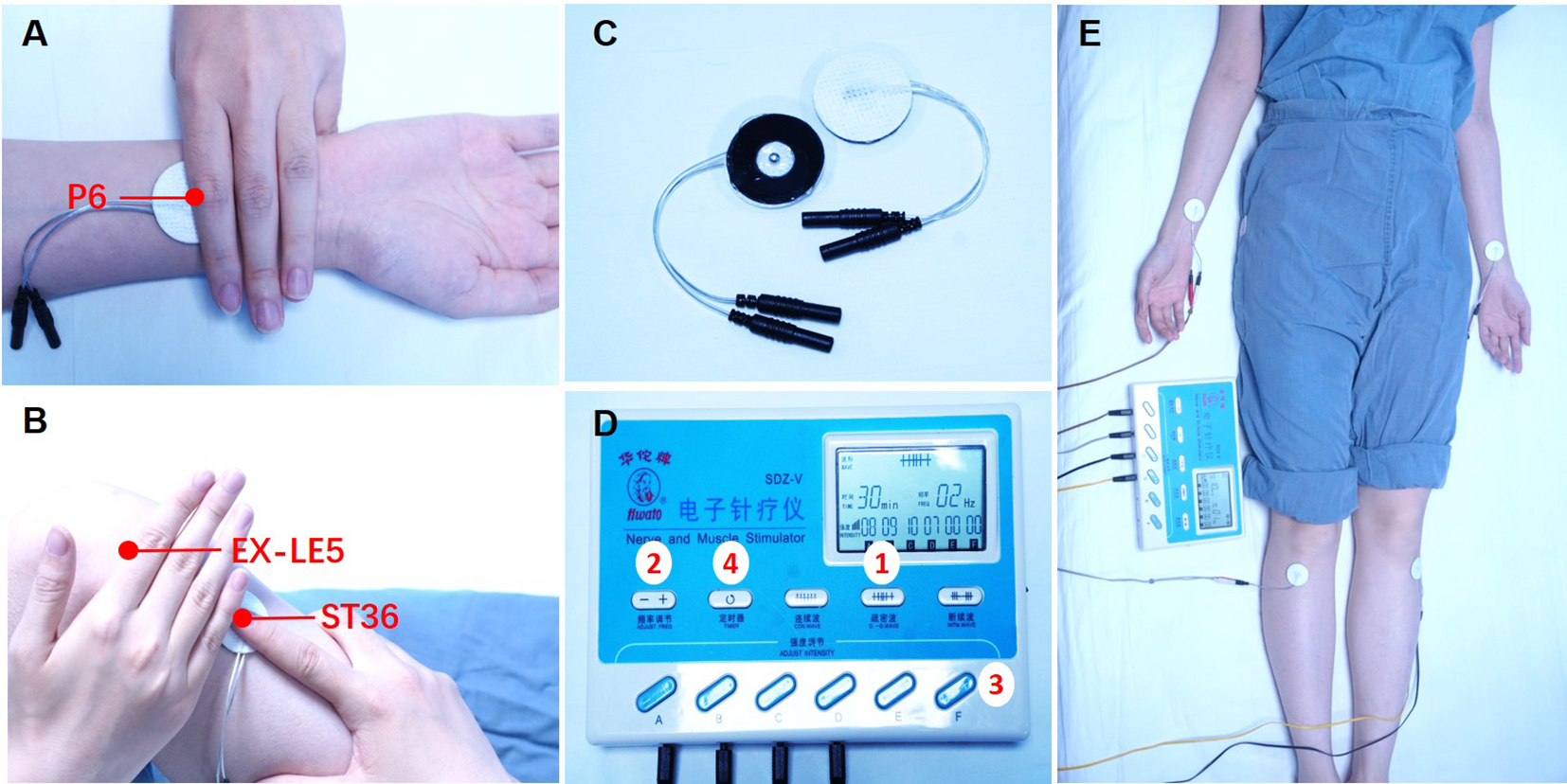


**eFigure 1. Standard Operating Procedure of the TEAS Treatment**

Acupuncture points (A) Neiguan (P6) and (B) Zusanli (ST36) are respectively located in bilateral arms and legs. P6 is located at 2 inches above palm wrist transverse striation, between the palm long tendon and the temporal flexor tendon. EX-LE5 is located in the lateral pit of the patella and patellar ligament. ST36 is located at four-finger widths (from the index finger, the little finger) down EX-LE5, and one-finger space behind the anterior border of tibia. (C) The electrode pads (Circular 30mm; TUORen, Henan, China) used for TEAS have a single conductive emboss and two connecting wires. (D) Interveners is connected electrode slices to electrical stimulator (TEAS stimulator, SDZ-V; Hwato, China) and adjusted with parameters: ① select the distant-dense wave, ② set the frequency of 2/10Hz, ③ adjust the intensity, the patient’s maximum tolerance, ④ and set treatment time for 30 minutes. (E) The patients in the both groups received identical settings and manipulation, except that the wire in Sham group between electrode pads and TEAS stimulator was cut so that no current could be delivered.


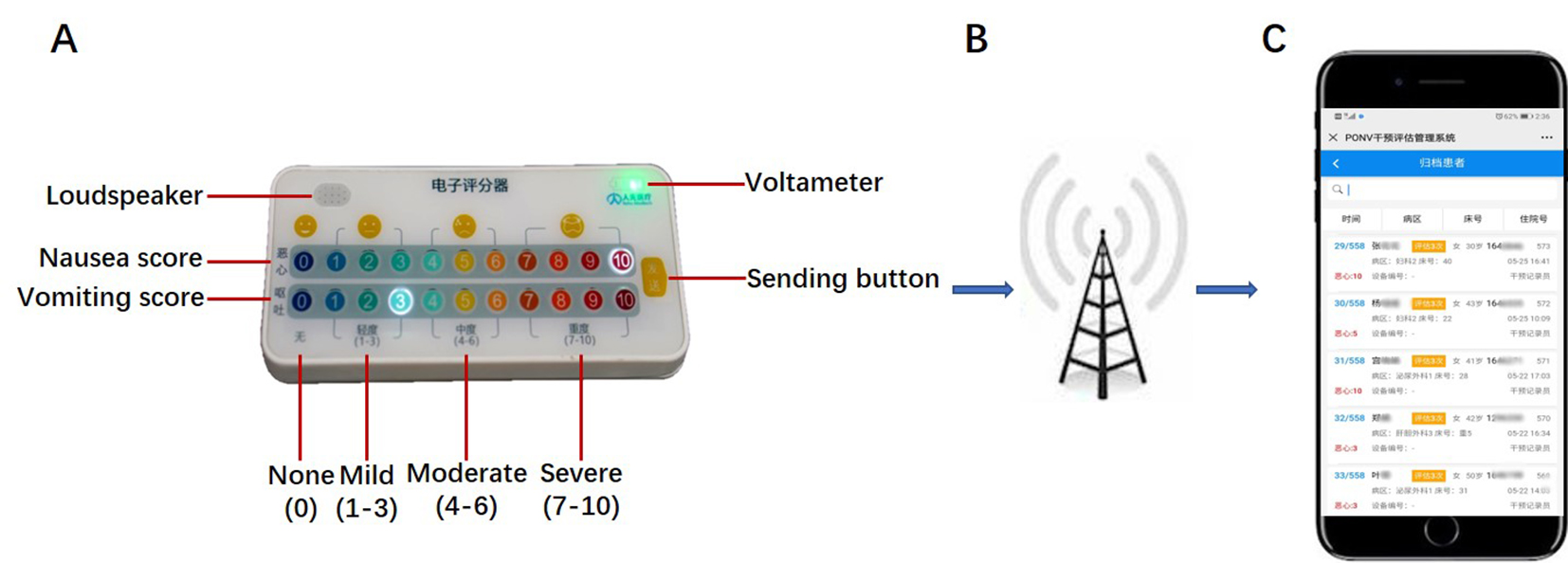


**eFigure 2. Workflow of the electronic patient self-reported scale**

(A) Electronic patient self-reported scale. Once PONV happens, patients select the nausea and vomiting scores and then press sending button. Voice reminder will indicate the success of information transmission. (B) The signal station in wards receives and sends PONV information to (C) mobile client management system. The evaluation times and PONV scores were recorded in real time.


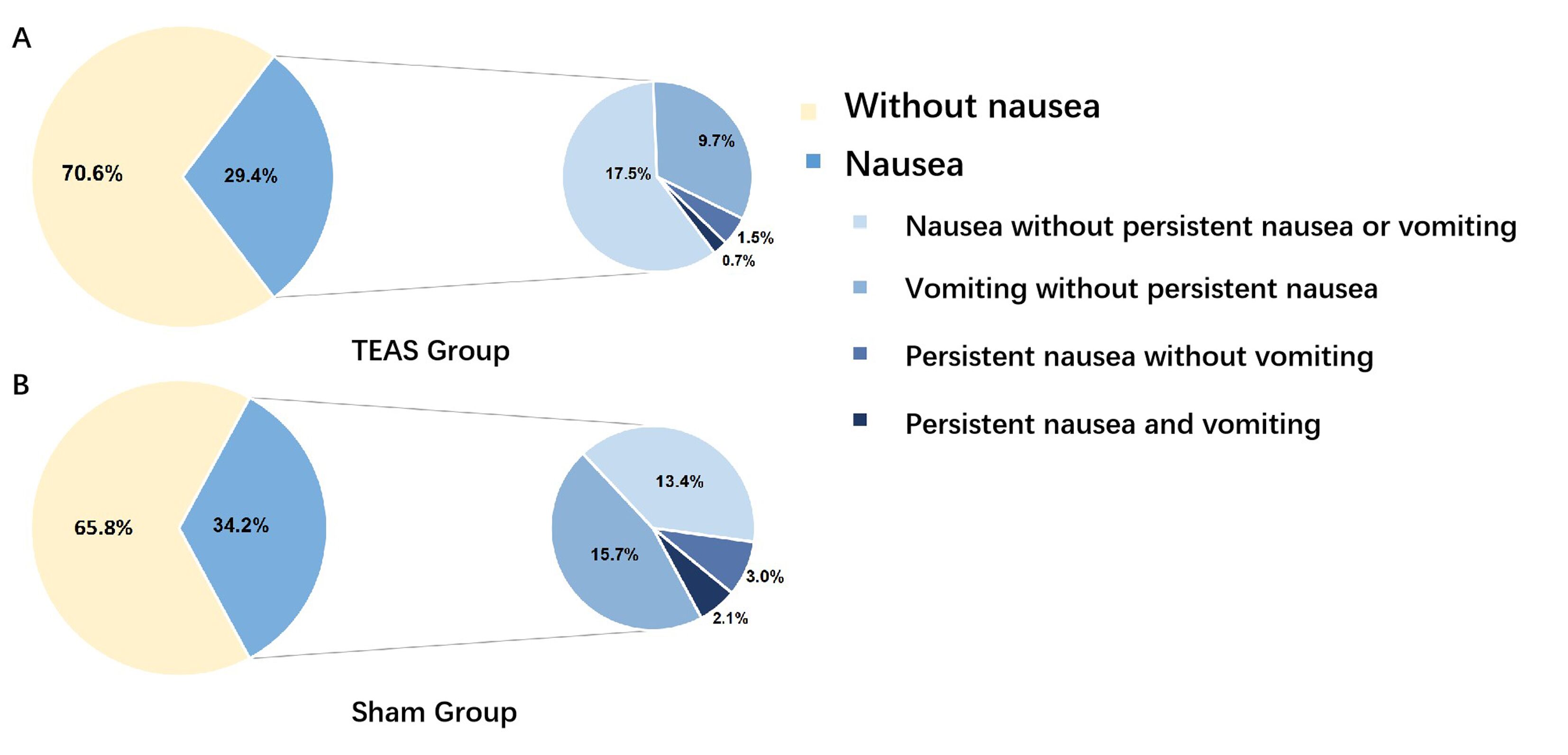


**eFigure 3. The population distribution in (A) TEAS and (B) Sham groups.**


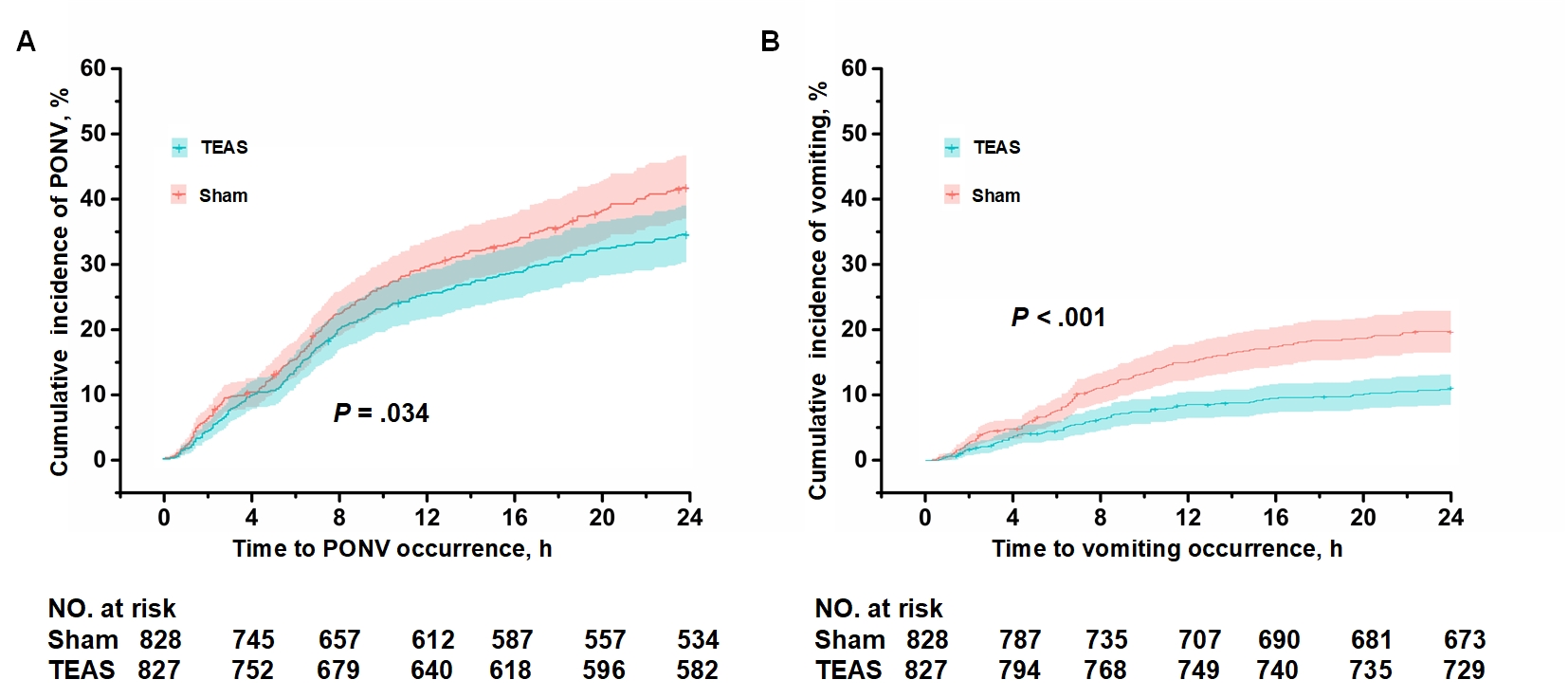


**eFigure 4. The postoperative 24 h cumulative (A) PONV and (B) vomiting incidences between TEAS and Shamgroup.**

**eTable 1.** **24 h QoR-40 Scores. Data are presented as median (IQR).**

|  | Physical  comfort | Emotional  state | Body independence | Psychological support | Pain |
| --- | --- | --- | --- | --- | --- |
| TEAS | 53.0  (49.0, 56.0) | 40.0  (37.0, 42.0) | 20.5  (15.0, 22.0) | 35.0  (35.0, 35.0) | 33.0  (31.0, 34.0) |
| Sham | 52.0  (47.0, 55.0) | 39.0  (36.0, 42.0) | 20.0  (14.0, 22.0) | 35.0  (33.0, 35.0) | 33.0  (30.0, 34.0) |
| *P* value | .003 | .001 | .302 | .002 | .012 |
|  |  |  |  |  |  |
| PONV | 48.0  (40.0, 51.8) | 38.0  (31.3, 40.0) | 18.0  (14.0,21.0) | 35.0  (28.0, 35.0) | 32.0  (28.0, 34.0) |
| Non-PONV | 54.0  (51.0, 57.0) | 40.0  (38.0, 43.0) | 21.0  (14.0, 23.0) | 35.0  (35.0, 35.0) | 34.0  (32.0, 35.0) |
| *P* value | <.001 | <.001 | .001 | <.001 | <.001 |
|  |  |  |  |  |  |
| Vomiting | 44.0  (31.3, 48.0) | 36.0  (24.3, 40.0) | 18.0  (15.0, 22.0) | 31.0  (25.0, 35.0) | 30.0  (27.0, 33.0) |
| Non-Vomiting | 53.0  (50.0, 56.0) | 40.0  (38.0, 42.0) | 21.0  (14.0, 22.0) | 35.0  (35.0, 35.0) | 33.0  (31.0, 35.0) |
| *P* value | <.001 | <.001 | .136 | <.001 | <.001 |

**Abbreviations: IQR, interquartile range; QoR-40,** **Quality of Recovery–40**

**eTable 2.** **The application of** **Prophylaxis antiemetic. Data are presented as number (%).**

|  | **TEAS**  **(n = 827)** | **Sham**  **(n = 828)** | ***P* value** |
| --- | --- | --- | --- |
| **Dexamethasone (only)** | 0 (0) | 0 (0) | / |
| **Palonosetron (only)** | 24 (2.9) | 21 (2.5) | 0.209 |
| **Tropisetron (only)** | 33 (4.0) | 49 (5.9) | 0.070 |
| **Dexamethasone+ Palonosetron** | 407 (49.2) | 417 (50.4) | 0.640 |
| **Dexamethasone+ Tropisetron** | 363 (43.9) | 341 (41.2) | 0.265 |
