# Supplementary material for: Transcutaneous Electrical Acupoint Stimulation Decreases the Incidence of Postoperative Nausea and Vomiting After Laparoscopic Non-gastrointestinal Surgery: A Multi-Center Randomized Controlled Trial
Source: Front Med (Lausanne). 2022 Mar 14;9:766244. doi: 10.3389/fmed.2022.766244 (PMC8964119; doi:10.3389/fmed.2022.766244)
Supplement: Supplementary file 2 [file Table_1.doc]

**Clinical Trial Protocol：**

**Prevention and Treatment of Postoperative Nausea and Vomiting by Transcutaneous electrical acupoint stimulation: a Multi-center, Evaluator-blinded, Randomized and Controlled Study**

**RESEARCH PROTOCOL**

Version 5.0 September 20, 2019

ClinicalTrials.gov identifier: NCT04043247

Project date: September 2019 -- February 2021

**Corresponding Author: Qiang Wang**

Department Director, Clinical Professor, Chief Doctor;

Department of Anesthesiology, the First Affiliated Hospital of Xi’an Jiaotong University

277 Yanta West Road, Xi’an 710061, Shaanxi, China

Phone & Fax: 86-29-8532-3646; Email: dr.wangqiang@mail.xjtu.edu.cn

**SYNOPSIS**

1. SUMMARY 1

2. SCIENTIFIC RATIONALE AND GENERAL DESCRIPTION OF RESEARCH 1

3. RESEARCH OBJECTIVES 2

4. RESEARCH DESIGN 2

4.1 Type of study 2

4.2 Primary and Secondary outcomes 2

4.2.1 Baseline information 2

4.2.2 Primary outcome 2

4.2.3 Secondary outcome 2

4.3. Description of research methodology 2

4.3.1 Recruitment 3

4.3.2 Screening and intervention 3

4.3.3 Pharmacologic and non-pharmacologic intervention 3

4.4 Description of measures taken to reduce and avoid bias 4

4.4.1 Randomization 4

4.4.2 Blinding method 4

4.4.3 Follow-up 4

4.5 Summary table of chronology of research and examinations conducted 4

4.6 Description of temporary stop rules 5

5. PATIENTS SELECTION AND EXCLUSION 5

5.1 Inclusion criteria 5

5.2 Exclusion criteria 5

6. PERIOPERATIVE MANAGEMENT FOR PATIENTS 5

7. Data collected 6

8. SAFETY ASSESSMENT 6

9. STATISTICAL ANALYSIS 6

10. QUALITY CONTROL 7

11. REQUEST FOR THE ETHICAL COMMITTEE 7

12. REFERENCES 7

**GENERAL INFORMATION**

# 1. SUMMARY

**Introduction:** Postoperative nausea and vomiting (PONV) is among the most common postoperative complications, which gives patients extremely afflictive medical experience and sometimes intractable side-effect, leading to prolonged hospitalization, increased medical expenses and lower surgical satisfaction. PONV prophylaxis has been included in enhanced recovery after surgery (ERAS) management strategy. Even though the incidence of PONV can be decreased by using less of opioid and inhalational anesthetics and/or using antiemetic drugs, its occurrence is still high up to 20% in high-risk patients. Complementary to the antiemetic drugs, non-pharmacotherapy acupuncture treatment, represented by transcutaneous electrical acupoint stimulation (TEAS), has been used to prevent PONV occurrence. However, its effectiveness in preventing PONV has not been established yet due to small sampling size and not well controlled in the previous studies.

**Hypothesis:** TEAS treatment based on standardized antiemetic prophylaxis significantly lowers PONV incidence than the Sham treatment.

**Primary outcome:** PONV incidence

**Secondary outcomes:** PONV quantitative indicators including the time and VAS score of first nausea and vomiting, the times and VAS scores of each nausea and vomiting within 24 h after surgery; ERAS items including the 40-item Quality of Recovery (QoR-40) within 24 h, PONV related complications.

**Methods:** A multicenter, prospective, evaluator-blinded, parallel-group, randomized controlled trial (RCT) is administered in Sham or TEAS treatment on laparoscopic non-gastrointestinal surgery patients.

**Number of patients:** The number of patients to be included 1655 cases in total.

**Inclusion criteria:** 1) selective laparoscopic non-gastrointestinal surgery under general anesthesia; 2) age 18–50 years, BMI 15–40 kg/m^2^, ASA class I–III; 3) Apfel score ≥3 points; 4) ability to understand, sign informed consent and coordinate intervention and evaluation.

**Exclusion criteria:** 1) pregnancy and breast-feeding patients; 2) TEAS contraindications: skin allergy, breakage, infection or itching of test points, allergic to adhesive tape, pacemaker implanted; 3) history of alcohol, opioids or other drug abuse; 4) likely admission to ICU after surgery; 5) participation in other clinical studies within 3 months before admission to this study.

# 2. SCIENTIFIC RATIONALE AND GENERAL DESCRIPTION OF RESEARCH

PONV characterized as nausea, retching or vomiting or any these symptoms in combination after surgery, is one of the most common complaints after surgery with an overall incidence of 20%–30%(1, 2). Nausea gives patients extremely afflictive medical experience, induces the stomach discomfort including the feeling of keck and poor appetite, and even causes dizziness and headache. Vomiting increases the potential risks of electrolyte imbalances, pain, incision dehiscence or hernia, dehydration, esophageal damage or airway aspiration(3). The incidence in high-risk patients (more than 3 scores), classified by Apfel simplified score which is based on four independent risk predictors (female gender, nonsmoker, history of motion sickness or PONV, postoperative opioid use), is 61%–79%(4). With The combination of 2–3 kinds of antiemetic drugs for PONV, the incidence of high-risk patients had a significant decline, however, still as high as 20%(5). What's worse is that the actual incidence of PONV is even higher due to the loss to follow-up bias by traditional paper diaries(6). The multiple channel and sites of PONV could not be blocked by the single locus of antiemetic drugs(7). It is difficult to further reduce the incidence of PONV by adding different kinds or dosages of drugs for the remaining uncovered PONV sites and the saturation of receptors(8), while drug-related side effects are increasing gradually(9)，such as headaches, xerostomia, abnormal liver function, extrapyramidal reactions and other related side effects(9-11).

Complementary to the antiemetic drugs, non-pharmaceutical therapy including acupuncture provides considerable advantages in wide action range and non-toxic effects, which has been widely used in postoperative pain analgesia and gastrointestinal function rehabilitation(12, 13). Acupuncture, a safe and effective non-pharmacotherapy, can directly or indirectly inhibits the emesis center or regulates gastrointestinal function through multiple regulatory mechanism, such as modulating gastric motility through somatovisceral reflex, influencing the endogenous opioid system and serotonin transmission(14-17). Transcutaneous electrical acupoint stimulation (TEAS) with transcutaneous electrical nerve stimulation applied to acupuncture points has been used to prevent PONV occurrence(18, 19). However, its effectiveness in preventing PONV has not been established yet due to small sampling size and not well controlled in the previous studies(20). Therefore, the large sample size, multicenter, evaluator-blinded, and randomized controlled study were carried out to verify the effectiveness of TEAS in reducing the incidence of PONV.

# 3. RESEARCH OBJECTIVES

This trial aims to assess the preventive effectiveness of transcutaneous electrical acupoint stimulation (TEAS) on the incidence of PONV in high-risk surgical patients.

# 4. RESEARCH DESIGN

# 4.1 Type of study

This work is a multicenter, prospective, evaluator-blinded, parallel-group, RCT. Using Crabyter scientific research system (Xinyu Information Technology Co., Ltd), the enrolled patients will be randomly divided into Sham and TEAS groups in a 1:1 ratio, with four patients regarded as a block, and obtain the random number.

# 4.2 Primary and Secondary outcomes

# 4.2.1 Baseline information

Demographic information (age, sex, height, weight, education, occupation, etc.), preoperative information (biochemical indicators, comorbidities, anxiety scores, etc.), surgical and anesthetic information (operation name and period, amount of access, RASS score, dosage of analgesics and antiemetics, etc.) will be compared in the two groups.

# 4.2.2 Primary outcome

In this study, at least once nausea, retching or vomiting after operation, or any combination of the above symptoms within 24 h after surgery is taken as the diagnostic criteria of PONV, thereby setting PONV incidence 24h as a primary outcome.

# 4.2.3 Secondary outcome

Secondary research indicators are PONV quantitative indicators and ERAS-related items. PONV quantitative indicators include the time and visual analogue scale (VAS) score of first PONV, the cumulative times and the highest VAS score of nausea and vomiting within 24 h after surgery. ERAS items include the 40-item Quality of Recovery (QoR-40) within 24 h, PONV related complications and postoperative hospital stay. The specific contents of QoR-40 score include five aspects: physical comfort (12 items, 60 scores), emotional state (9 items, 45 scores), physical independence (5 items, 25 scores), psychological support (7 items, 35 scores) and pain (7 items, 35 scores)(21). PONV related complications are dizziness and headache, electrolyte imbalances and pain.

# 4.3 Description of research methodology

# 4.3.1 Recruitment

The study will take the First Affiliated Hospital of Xi’an Jiaotong University as the principle research center, allying with 11 Sub-Centers, Affiliated Hospital of Shaanxi University of Chinese Medicine, First Affiliated Hospital of Henan University of Chinese Medicine, First Affiliated Hospital of Henan University of Science and Technology, First Hospital of Hunan University of Chinese Medicine, General Hospital of Ningxia Medical University, Henan Provincial People's Hospital, Hunan Provincial People's Hospital, Second Hospital of Shanxi Medical University, Shanxi Provincial Hospital of Traditional Chinese Medicine, Shuguang Hospital Affiliated to Shanghai University of Traditional Chinese Medicine, Tianjin Third Central Hospital. The study will recruit the patients scheduled for laparoscopic non-gastrointestinal surgery under general anesthesia in 12 hospitals above between September 2019 and February 2021.

### Prescreening and rescreening

All investigators will be trained and will follow the trial protocol. Researchers will assess whether the scheduled patient meets the criteria one day before the surgery. Informed consent will be signed if the patient meets the eligibility, and then make an evaluation of self-rating anxiety scale (SAS). Patients’ eligibility for recruitment will be reassessed after extubation. The patients given conversion to open laparotomy, occurring skin allergy, breakage, infection or itching of test points, refusing to participate or unplanned admission to ICU will be excluded from the trial. If they are eligible, randomization will be performed. Using Crabyter scientific research system (Xinyu Information Technology Co., Ltd) for block randomization, the enrolled patients will be randomly divided into Sham and TEAS groups in a 1:1 ratio (with a block size of 4) to verify the reliability of TEAS in PONV prevention. The enrolled patients will be assessed by Richmond Agitation-Sedation Scale (RASS).

### 4.3.3 Pharmacologic and non-pharmacologic intervention

Attending anesthesiologists administrated PONV prophylaxis in operation room. Dexamethasone 5 mg IV and the second-generation 5-hydroxytryptamine 3 (5-HT_3_) receptor antagonists palonosetron 0.075 mg are administrated before anesthesia induction as the first choice. When palonosetron was not available at some hospitals, patients received dexamethasone 5 mg (before induction) combined with tropisetron 2 mg (at the end of surgery) instead. According to the criteria of WHO acupuncture points, Neiguan (P6) and Zusanli (ST36) will be selected in this study. As shown in the Supplementary Figure 1, these acupoints are far from the operation site. P6 is located at 2 inches above palm wrist transverse striation, between the palm long tendon and the temporal flexor tendon. ST36 is located at 3 inches below EX-LE5 (the lateral pit of the patella and patellar ligament), and one transverse finger outward lateral tibia. The electrode slice has a single conductive emboss and two connecting wires, thereby enabling non-invasive, accurate and convenient stimulation. The TEAS treatments will be scheduled to conduct twice in total, respectively during anesthesia recovery in PACU (to be awake and calm, RASS = 0) and in the next-morning of surgical ward for 30 minutes.

**TEAS group:** When patients recover from anesthesia status to a degree of the Steward recovery score ≥ 4 in PACU and the endotracheal tubes are withdrawn, the first TEAS treatment will be performed. The bilateral P6 and ST36 will be pressed to achieve a tingling sensation called “de qi”, and electrode slices will be stuck and fixed on those acupoints. Interveners will connect electrode slices to electrical stimulator and select the distant-dense wave with the frequency of 2/10Hz. After adjusting the intensity to the patient’s maximum tolerance, he/she will be treated for 30 minutes. Another TEAS treatment will be performed between 9 am and 12 am in the ward on the first postoperative day; meanwhile, patients will receive routine health education including illness, diet, exercise and psychological considerations.

**Sham group:** Electrode slices will be stuck at the same acupoints as in TEAS group. The interveners will connect electrode slices to stimulator through the internally broken wires and set the same treatment procedure with key sound. The treatment will last for 30 minutes without adjusting to the patient’s maximum tolerance intensity. To protect the rights of patients, the interveners will receive the same health education as it in TEAS group in the morning of the first postoperative day.

## 4.4 Description of measures taken to reduce and avoid bias

### 4.4.1 Randomization

Crabyter scientific research system (Xinyu Information Technology Co., Ltd) will be used for block randomization, with four patients comprising each block. Each center will compete into block to enroll subjects. A block group will be randomly allocated into TEAS group or Sham group at a ratio of 1: 1, with four patients regarded as one block. This random method ensures that the number of patients in two groups will be essentially equal with highly efficient enrollment.

### 4.4.2 Blinding method

Patients will be blinded when being allocated and cannot detect any difference in the entire procedures. This procedure will be the same in both groups, except that the connecting wires inside the stimulators will be broken in the Sham group. Therapeutic evaluators will be blinded in the process of allocation, screening, intervention treatment and statistical analysis. To prevent the leakage of allocation, evaluator will not play any other roles in the study, and we will apply spatial and temporal isolation for interventions and evaluations. Interventions will be conducted in PACU and ward, whereas evaluations will be performed only in the ward. Interventions and evaluations will be performed during different periods in ward. Interventions will be conducted once a day between 9 am and 12 am in the ward, whereas evaluations will be performed after 12 am. When the patient is randomized, an evaluator will complete the entire process of evaluation in a blinded intervention progress. A random administrator will be selected from principle investigator research center. This administrator will not play any other roles in the study. The external statistical experts will be blinded to allocation, and the statistical calculations will be independently conducted.

### 4.4.3 Follow-up

Electronic patient self-reported scale (Jiangsu Rehn Medtech Technology Co., Ltd) will be applied to evaluate and record the occurrence of PONV in real time, including the time to each PONV and corresponding VAS score. The VAS is a simple-to-use measure to evaluate intensity of PONV. It’s a 10-point numerical rating scale with 0 = “no nausea or vomiting” and 10 = “worst nausea imaginable and vomiting”. The cut points on the PONV VAS have been recommended: mild PONV (< 4), which has little impact on daily activities and does not affect sleep; severe PONV (> 7), which results in inability to sleep and seriously hinders daily life; moderate PONV, whose clinical influence intervenes between the two above. When PONV happens at any time, patients or their caregivers spontaneously assess the severity of PONV with VAS and then send it to the central server through the electronic scale. The evaluator port harvests PONV information immediately so that it can be managed timely. To ensure a blinded fashion, both groups will be evaluated by direct questioning in the ward after 12 am at the 1st and 2nd postoperative day in ward. Persistent nausea was defined as continuous nausea lasting over 5 min. If the patients are discharged on the second day postoperative, they will be assessed by telephone. The postoperative 30-day information about delayed curative efficacy, complications and adverse events will be obtained through telephone follow-ups and electronic medical record system.

### 4.5 Summary table of chronology of research and examinations conducted

**Table 1. Clinical Research Process**

|  | **BOD**  **1** | **OD**  **OR** | **OD**  **PACU** | **POD**  **1** | **POD**  **2** | **POD**  **30** |
| --- | --- | --- | --- | --- | --- | --- |
| **preliminary screening** | √ |  |  |  |  |  |
| **Informed consent** | √ |  |  |  |  |  |
| **SAS** | √ |  |  |  |  |  |
| **Rescreening** |  |  | √ |  |  |  |
| **Randomization** |  |  | √ |  |  |  |
| **RASS** |  |  | √ |  |  |  |
| **Intervention** |  |  | √ | √ |  |  |
| **Evaluation** |  |  |  | √ | √ | √ |

√ : routinely performed, BOD 1: 1 day before operation, OD: operation day, OR: operation room, PACU: post anesthesia care unit, POD (1, 2, 30): (1, 2, 30) day after operation

### 4.6 Description of temporary stop rules

**Exit criteria:** The exit criteria includes the following: 1) patients who exit and terminate observation due to serious adverse events; 2) have other diseases and are unwilling to continue treatment; 3) want to exit for any reason not related to the study; 4) mistakenly included; 5) with poor compliance and unable to cooperate with the researchers to complete the intervention or cannot provide important assessment information.

### 5. PATIENTS SELECTION AND EXCLUSION

### 5.1 Inclusion criteria

These criteria include the following: 1) selective laparoscopic non-gastrointestinal surgery under general anesthesia; 2) age 18–50 years old, BMI 15–40 kg/m^2^, ASA class I–III; 3) PONV risk prediction score Apfel ≥ 3 points; 4) ability to understand, sign informed consent and coordinate intervention and evaluation.

### 5.2 Exclusion criteria

These criteria include the following: 1) pregnancy and breast-feeding patients; 2) TEAS contraindications: skin allergy, breakage, infection or itching of test points, allergic to adhesive tape, pacemaker implanted; 3) history of alcohol, opioids or other drug abuse; 4) likely admission to ICU after surgery; 5) participation in other clinical studies within 3 months before admission to this study.

### 6. PERIOPERATIVE MANAGEMENT FOR PATIENTS

Attending anesthesiologists administrated PONV prophylaxis in operation room. Dexamethasone 5 mg IV and the second-generation 5-hydroxytryptamine 3 (5-HT_3_) receptor antagonists palonosetron 0.075 mg are administrated before anesthesia induction, or dexamethasone 5 mg and tropisetron 2mg are respectively administrated before anesthesia induction and at the end of surgery. Patients will be inducted with propofol (2 mg/kg), sufentanil (0.3 μg/kg) and cisphenyl sulfatracurium (0.15 mg/kg), and intubated. Anaesthesia maintenance will be performed using remifentanil (0.1 μg/kg/h), cisphenyl sulfatracurium (0.1 mg/kg/h), dexmedetomidine (initial dose 1 μg/kg for 10 min, maintenance dose 0.4 μg/kg/h) and sevoflurane (oxygen flow of 2 L/min and a dial setting of 1%) and/or propofol (4–8 mg/kg/h) as necessary to keep BIS 40-60 throughout surgery. All patients will receive identical multimodal analgesia in both arms to keep the postoperative pain numerical score less than 4 points. Patients will be rescued once PONV score >7. If it is not exceeded 6 h after the first 5-HT_3_ administration, propofol 20 mg IV or haloperidol 0.5–2 mg IV will be administrated. If it is exceeded 6 h after the first 5-HT_3_ administration, tropisetron 0.5 mg or ondansetron 2 mg IV will be administrated.

### 7. Data collected

The CRF table will be entered on Crabyter scientific research system (Xinyu Information Technology Co., Ltd), respectively by interveners and evaluators. It will include preoperative and postoperative screening forms, the informed consent form, anesthesia，antiemetic and analgesic record forms, postoperative intervention forms, postoperative evaluation forms, adverse event registration, exit registration forms, etc. Electronic patient self-reported scale (Jiangsu Rehn Medtech Technology Co., Ltd) was used to evaluate and record the occurrence of PONV, including time and corresponding severity. After completing the study, data administrator will retrieved all data in excel format. The postoperative 30-day complications and adverse events were obtained through telephone follow-ups and were stored in the electronic medical record system.

### 8. SAFETY ASSESSMENT

As TEAS are non-invasive, patients usually have a good tolerance, and the possible adverse reactions are mostly uncomfortable caused by electric stimulation, redness and swelling of electrode adhesion site, while very few patients may appear as hypoglycemia due to active gastrointestinal movement caused by acupoint stimulation. Most adverse reactions are mild and disappear after cease of electrical stimulation. No adverse reactions have been observed in the pre-experiment, and no serious adverse reactions have been reported in previous studies. If occurring from the day patients enrolled to 30 days after the operation, adverse events will be processed and recorded in time, and reported to the principle investigator as well as the director of ethics committee of the hospital in 24 h.

### 9. STATISTICAL ANALYSIS

**Sample size estimation and data analysis：**In previous studies, the PONV incidence was reported to be approximately 40%, and the PONV incidence was reduced by 7% with the TEAS treatment(19). With a significance set at 0.05, power set at 80%, the sample size required to detect differences was 742 patients in each group calculated with the Pass 11 software (NCSS, LLC. Kaysville, Utah, USA). Taking into account a loss-to-follow-up rate of about 10%, 1634 patients in total were enrolled to meet the minimum sample size requirement.

**Sample distribution:** Describes the size and loss rate of each data set, and provides detailed reasons for loss.

**Data comparability:** Preoperative baseline information, surgical information, anesthetic and antiemetic drug usage are compared between the two groups.

**Validity analysis:** The primary and secondary outcomes of the two groups are compared.

**Safety analysis:** Chi-square statistical tests are performed for the number and incidence of adverse events.

According to the intentionality analysis ITT principle, all subjects are included even if they have undergone in part of the trial process. At the same time, the efficacy and safety indicators will be included into the PP data set conformed to the schemed set, and then statistically analyzed in strict accordance with the scheme. Continuous variables will be described by means (SDs) and median (IQR), the differences between the two groups will be compared using the independent-sample T test. Categorical variables will be described by numbers (percentages), and chi-square test and Fisher's accuracy test will be used to test the incidence. Nonparametric rank sum test will be used for the grade data. *P value*<.05 will be considered statistically significant. All statistical analyses will be performed using SPSS software (version 25.0, IBM Corporation).

### 10. QUALITY CONTROL

**Quality control system (QC)：**In this study, two quality control levels will be established. In the first level, the internal QC inspectors who will not participate in the implementation will be selected from the department. The second level quality control will be monitored by a hospital quality control officer. The quality control manager will examine, correct, and report the violative behavior of the study protocol, and check if the data record is true, timely, accurate and complete.

### 11. REQUEST FOR THE ETHICAL COMMITTEE

The study was approved by the Ethics Committee of the First Affiliated Hospital of Xi’an Jiaotong University (approval code XJTU1AF2019LSK-084) and registered with Clinical Trials Registry (NCT04043247). According to the Declaration of Helsinki requirements of informed consent, all subjects will voluntarily sign the informed consent form.

### 12. REFERENCES

1. Habib AS, Gan TJ. Postoperative nausea and vomiting: then & now. *Anesthesia and analgesia* (2012) 115(3):493-5. Epub 2012/08/23. doi: 10.1213/ANE.0b013e318254285e.

2. Rusch D, Eberhart LH, Wallenborn J, Kranke P. Nausea and vomiting after surgery under general anesthesia: an evidence-based review concerning risk assessment, prevention, and treatment. *Dtsch Arztebl Int* (2010) 107(42):733-41. Epub 2010/11/17. doi: 10.3238/arztebl.2010.0733.

3. Schmitt ARM, Ritto FG, de Azevedo J, Medeiros PJD, de Mesquita MCM. Efficacy of Gastric Aspiration in Reducing Postoperative Nausea and Vomiting After Orthognathic Surgery: A Double-Blind Prospective Study. *J Oral Maxillofac Surg* (2017) 75(4):701-8. Epub 2016/11/07. doi: 10.1016/j.joms.2016.10.002.

4. Kovac AL. Update on the management of postoperative nausea and vomiting. *Drugs* (2013) 73(14):1525-47. Epub 2013/09/24. doi: 10.1007/s40265-013-0110-7.

5. Apfel CC, Roewer N. [Postoperative nausea and vomiting]. *Anaesthesist* (2004) 53(4):377-89; quiz 90-1. Epub 2004/06/12. doi: 10.1007/s00101-004-0662-8.

6. Bandarian-Balooch S, Martin PR, McNally B, Brunelli A, Mackenzie S. Electronic-Diary for Recording Headaches, Triggers, and Medication Use: Development and Evaluation. *Headache* (2017) 57(10):1551-69. Epub 2017/09/20. doi: 10.1111/head.13184.

7. Chandrakantan A, Glass PS. Multimodal therapies for postoperative nausea and vomiting, and pain. *Br J Anaesth* (2011) 107 Suppl 1:i27-40. Epub 2011/12/22. doi: 10.1093/bja/aer358.

8. Cao X, White PF, Ma H. An update on the management of postoperative nausea and vomiting. *J Anesth* (2017) 31(4):617-26. doi: 10.1007/s00540-017-2363-x.

9. Kovac A. Prevention and treatment of postoperative nausea and vomiting. *Drugs* (2000) 59(2):213-43. doi: 10.2165/00003495-200059020-00005.

10. Kovac AL. Comparative Pharmacology and Guide to the Use of the Serotonin 5-HT3 Receptor Antagonists for Postoperative Nausea and Vomiting. *Drugs* (2016) 76(18):1719-35. Epub 2016/12/19. doi: 10.1007/s40265-016-0663-3.

11. Gan T, White P, Scuderi P, Watcha M, Kovac A. FDA "black box" warning regarding use of droperidol for postoperative nausea and vomiting: is it justified? *Anesthesiology* (2002) 97(1):287. doi: 10.1097/00000542-200207000-00059.

12. Chen J, Zhang Y, Li X, Wan Y, Ji X, Wang W, et al. Efficacy of transcutaneous electrical acupoint stimulation combined with general anesthesia for sedation and postoperative analgesia in minimally invasive lung cancer surgery: A randomized, double-blind, placebo-controlled trial. *Thorac Cancer* (2020). Epub 2020/02/18. doi: 10.1111/1759-7714.13343.

13. Zhou D, Hu B, He S, Li X, Gong H, Li F, et al. Transcutaneous Electrical Acupoint Stimulation Accelerates the Recovery of Gastrointestinal Function after Cesarean Section: A Randomized Controlled Trial. *Evid Based Complement Alternat Med* (2018) 2018:7341920. doi: 10.1155/2018/7341920.

14. Stoicea N, Gan TJ, Joseph N, Uribe A, Pandya J, Dalal R, et al. Alternative Therapies for the Prevention of Postoperative Nausea and Vomiting. *Front Med (Lausanne)* (2015) 2:87. Epub 2016/01/07. doi: 10.3389/fmed.2015.00087.

15. Wang C, Chen X, Xie PY. Electroacupuncture at PC6 or ST36 Influences the Effect of Tacrine on the Motility of Esophagus. *Evid Based Complement Alternat Med* (2014) 2014:263489. Epub 2014/05/09. doi: 10.1155/2014/263489.

16. Wang C, Zhou D, Shuai X, Liu J, Xie P. Effects and mechanisms of electroacupuncture at PC6 on frequency of transient lower esophageal sphincter relaxation in cats. *World journal of gastroenterology* (2007) 13(36):4873-80. doi: 10.3748/wjg.v13.i36.4873.

17. Streitberger K, Ezzo J, Schneider A. Acupuncture for nausea and vomiting: an update of clinical and experimental studies. *Auton Neurosci* (2006) 129(1-2):107-17. Epub 2006/09/05. doi: 10.1016/j.autneu.2006.07.015.

18. Fu C, Wu T, Shu Q, Song A, Jiao Y. Acupuncture therapy on postoperative nausea and vomiting in abdominal operation: A Bayesian network meta analysis. *Medicine (Baltimore)* (2020) 99(23):e20301. Epub 2020/06/06. doi: 10.1097/MD.0000000000020301.

19. Chen J, Tu Q, Miao S, Zhou Z, Hu S. Transcutaneous electrical acupoint stimulation for preventing postoperative nausea and vomiting after general anesthesia: A meta-analysis of randomized controlled trials. *Int J Surg* (2020) 73:57-64. Epub 2019/11/11. doi: 10.1016/j.ijsu.2019.10.036.

20. Gan TJ, Belani KG, Bergese S, Chung F, Diemunsch P, Habib AS, et al. Fourth Consensus Guidelines for the Management of Postoperative Nausea and Vomiting. *Anesthesia and analgesia* (2020) 131(2):411-48. Epub 2020/05/30. doi: 10.1213/ANE.0000000000004833.

21. Gornall BF, Myles PS, Smith CL, Burke JA, Leslie K, Pereira MJ, et al. Measurement of quality of recovery using the QoR-40: a quantitative systematic review. *Br J Anaesth* (2013) 111(2):161-9. Epub 2013/03/09. doi: 10.1093/bja/aet014.
