# Supplementary material for: Transcutaneous Electrical Acupoint Stimulation Decreases the Incidence of Postoperative Nausea and Vomiting After Laparoscopic Non-gastrointestinal Surgery: A Multi-Center Randomized Controlled Trial
Source: Front Med (Lausanne). 2022 Mar 14;9:766244. doi: 10.3389/fmed.2022.766244 (PMC8964119; doi:10.3389/fmed.2022.766244)
Supplement: Supplementary file 3 [file Table_2.doc]

CONSORT 2010 checklist of information to include when reporting a randomised trial

| Section/Topic | Item No | Checklist item | Reported on page No |
| --- | --- | --- | --- |
| Title and abstract | | | |
|  | 1a | Identification as a randomised trial in the title | 1 |
|  | 1b | Structured summary of trial design, methods, results, and conclusions (for specific guidance see CONSORT for abstracts) | 3 |
| Introduction | | | |
| Background and objectives | 2a | Scientific background and explanation of rationale | 4 |
|  | 2b | Specific objectives or hypotheses | 4 |
| Methods | | | |
| Trial design | 3a | Description of trial design (such as parallel, factorial) including allocation ratio | 4-5 |
|  | 3b | Important changes to methods after trial commencement (such as eligibility criteria), with reasons | N/A |
| Participants | 4a | Eligibility criteria for participants | 4-5 |
|  | 4b | Settings and locations where the data were collected | 6 |
| Interventions | 5 | The interventions for each group with sufficient details to allow replication, including how and when they were actually administered | 6 |
| Outcomes | 6a | Completely defined pre-specified primary and secondary outcome measures, including how and when they were assessed | 5,6 |
|  | 6b | Any changes to trial outcomes after the trial commenced, with reasons | N/A |
| Sample size | 7a | How sample size was determined | 7 |
|  | 7b | When applicable, explanation of any interim analyses and stopping guidelines | N/A |
| Randomisation: |  |  |  |
| Sequence generation | 8a | Method used to generate the random allocation sequence | 5 |
|  | 8b | Type of randomisation; details of any restriction (such as blocking and block size) | 5 |
| Allocation concealment mechanism | 9 | Mechanism used to implement the random allocation sequence (such as sequentially numbered containers), describing any steps taken to conceal the sequence until interventions were assigned | 5 |
| Implementation | 10 | Who generated the random allocation sequence, who enrolled participants, and who assigned participants to interventions | 5 |
| Blinding | 11a | If done, who was blinded after assignment to interventions (for example, participants, care providers, those assessing outcomes) and how | 5,6 |
|  | 11b | If relevant, description of the similarity of interventions | 6 |
| Statistical methods | 12a | Statistical methods used to compare groups for primary and secondary outcomes | 6 |
|  | 12b | Methods for additional analyses, such as subgroup analyses and adjusted analyses | 7 |
| Results | | | |
| Participant flow (a diagram is strongly recommended) | 13a | For each group, the numbers of participants who were randomly assigned, received intended treatment, and were analysed for the primary outcome | Figure1 |
|  | 13b | For each group, losses and exclusions after randomisation, together with reasons | Figure1 |
| Recruitment | 14a | Dates defining the periods of recruitment and follow-up | 5 |
|  | 14b | Why the trial ended or was stopped | N/A |
| Baseline data | 15 | A table showing baseline demographic and clinical characteristics for each group | Table1,2 |
| Numbers analysed | 16 | For each group, number of participants (denominator) included in each analysis and whether the analysis was by original assigned groups | 7 |
| Outcomes and estimation | 17a | For each primary and secondary outcome, results for each group, and the estimated effect size and its precision (such as 95% confidence interval) | Table 3,4 |
|  | 17b | For binary outcomes, presentation of both absolute and relative effect sizes is recommended | 7,8 |
| Ancillary analyses | 18 | Results of any other analyses performed, including subgroup analyses and adjusted analyses, distinguishing pre-specified from exploratory | 7,8 |
| Harms | 19 | All important harms or unintended effects in each group (for specific guidance see CONSORT for harms) | N/A |
| Discussion | | | |
| Limitations | 20 | Trial limitations, addressing sources of potential bias, imprecision, and, if relevant, multiplicity of analyses | 10 |
| Generalisability | 21 | Generalisability (external validity, applicability) of the trial findings | 10,11 |
| Interpretation | 22 | Interpretation consistent with results, balancing benefits and harms, and considering other relevant evidence | 8-10 |
| Other information | | |  |
| Registration | 23 | Registration number and name of trial registry | 3 |
| Protocol | 24 | Where the full trial protocol can be accessed, if available | Supplement 1 |
| Funding | 25 | Sources of funding and other support (such as supply of drugs), role of funders | 12 |

## STandards for Reporting Interventions in Clinical Trials of Acupuncture (STRICTA): extending the CONSORT Statement

| Section/Topic | Item No | Checklist item | Reported on page No |
| --- | --- | --- | --- |
| Acupuncture rationale | | | |
|  | 1a | Style of acupuncture (e.g. Traditional Chinese Medicine, Japanese, Korean, Western medical, Five Element, ear acupuncture, etc) | 1 |
|  | 1b | Reasoning for treatment provided, based on historical context, literature sources, and/or consensus methods, with references where appropriate | 4 |
|  | 1c | Extent to which treatment was varied | 6 |
| Details of needling | | | |
|  | 2a | Number of needle insertions per subject per session (mean and range where relevant) | 6 |
|  | 2b | Names (or location if no standard name) of points used (uni/bilateral) | 6 |
|  | 2c | Depth of insertion, based on a specified unit of measurement, or on a particular tissue level | 6 |
|  | 2d | Response sought (e.g. de qi or muscle twitch response) | 6 |
|  | 2e | Needle stimulation (e.g. manual, electrical) | 6 |
|  | 2f | Needle retention time | 6 |
|  | 2g | Needle type (diameter, length, and manufacturer or material) | N/A |
| Treatment regimen | | | |
|  | 3a | Number of treatment sessions | 6 |
|  | 3b | Frequency and duration of treatment sessions | 6 |
| Other components of treatment | | | |
|  | 4a | Details of other interventions administered to the acupuncture group (e.g. moxibustion, cupping, herbs, exercises, lifestyle advice) | N/A |
|  | 4b | Setting and context of treatment, including instructions to practitioners, and information and explanations to patients | N/A |
| Practitioner background | |  |  |
|  | 5 | Description of participating acupuncturists (qualification or professional affiliation, years in acupuncture practice, other relevant experience) | 5 |
| Control or comparator interventions | | | |
|  | 6a | Rationale for the control or comparator in the context of the research question, with sources that justify this choice | 6 |
|  | 6b | Precise description of the control or comparator. If sham acupuncture or any other type of acupuncture-like control is used, provide details as for Items 1 to 3 above. | 6 |
